# Advances in Gold Nanoparticles-Based Colorimetric Aptasensors for the Detection of Antibiotics: An Overview of the Past Decade

**DOI:** 10.3390/nano11040840

**Published:** 2021-03-25

**Authors:** Qurat ul Ain Zahra, Zhaofeng Luo, Rizwan Ali, Muhammad Imran Khan, Fenfen Li, Bensheng Qiu

**Affiliations:** 1 Hefei National Lab for Physical Sciences at the Microscale and the Centers for Biomedical Engineering, University of Science and Technology of China, Hefei 230026, China; rizwanali35@yahoo.com (R.A.); Imran_almani@yahoo.com (M.I.K.); lifenfen@ustc.edu.cn (F.L.); 2 Core Facility Center for Life Sciences, Department of Molecular Biology and Cell Biology, University of Sciences and Technology of China, Hefei 230026, China

**Keywords:** antibiotics detection, colorimetric aptasensors, aptamer-based sensors, antibiotic residue pollution, AuNPs-based aptasensors, aptasensors for antibiotics, colorimetric biosensors

## Abstract

Misuse of antibiotics has recently been considered a global issue because of its harmful effects on human health. Since conventional methods have numerous limitations, it is necessary to develop fast, simple, sensitive, and reproducible methods for the detection of antibiotics. Among numerous recently developed methods, aptasensors are fascinating because of their good specificity, sensitivity and selectivity. These kinds of biosensors combining aptamer with colorimetric applications of gold nanoparticles to recognize small molecules are becoming more popular owing to their advantageous features, for example, low cost, ease of use, on-site analysis ability using naked eye and no prerequisite for modern equipment. In this review, we have highlighted the recent advances and working principle of gold nanoparticles based colorimetric aptasensors as promising methods for antibiotics detection in different food and environmental samples (2011–2020). Furthermore, possible advantages and disadvantages have also been summarized for these methods. Finally, the recent challenges, outlook, and promising future perspectives for developing novel aptasensors are also considered.

## 1. Background and Introduction

Antibiotics, also known as antibacterial agents, may have natural or synthetic origin, e.g., microorganisms or numerous chemicals respectively. They are used in the prevention and treatment of bacterial infections [1]. Not only are they involved in saving patients’ lives, but they also play a key role in surgery and medicine-based advances [2]. Despite their overuse warnings, they are commonly overprescribed in many countries [3]. Using an overdose drives resistant bacterial evolution. In both developing and developed countries, they are widely used in livestock as growth supplements [4]. Subsequently, they are consumed by humans through ingesting those animal-derived foodstuffs [3]. Resistant bacterial species are also transmitted through the food chain to humans, causing severe infections leading to serious health conditions. The use of antibiotics in agricultural practices also have influences on environmental microbiome [4]. As a result, increased mortality and morbidity due to infections and compromised immune-system becomes virulent [3]. Thus, it is crucial to develop useful methods to cope with the long-term life-threatening effects caused by these antibiotic residues in the environment and food for their effectual monitoring [5,6].

At present, a variety of methods such as high-performance liquid chromatography (HPLC) [5,7,8,9,10], liquid chromatography-mass spectrometry [5,11,12,13,14], chemiluminescence (CL), immunoassays, electrochemical and capillary electrophoresis [5,15], gas chromatography-mass spectrometry [16], etc. are available for the specific detection of a variety of antibiotics. Liquid chromatography in combination with mass spectrometry (LC-MS) guarantees high resolution, target identification, selectivity, repeatability, sensitivity and application versatility [17]. The most common, currently-used approaches to confirm antibiotic residue presence are LC-MS, ELISA, and biosensor-based methods. All these methods are associated with some notable advantages (e.g., practicality and sensitivity, etc.) and disadvantages (e.g., unsatisfactory cost-effectiveness and slowness, etc.) [18] which do not impinge on the validity of the above reported methods.

## 2. Biosensors

**Figure 1:**
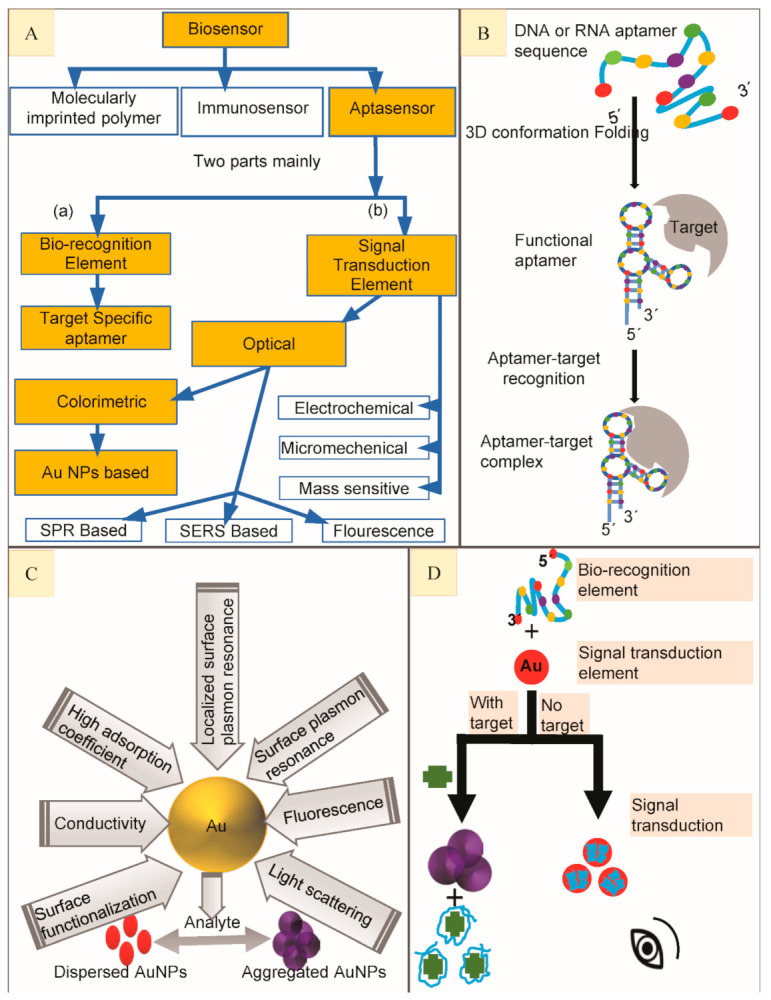
(**A**) Scheme to show main types of biosensors to highlight aptasensor, classification of aptasensors based on its major components to highlight Gold Nanoparticles (AuNPs) based colorimetric aptasensors. (**B**) Generalized depiction of an aptamer library folding to form secondary structure in order to bind with high affinity target molecule to finally make aptamer-target complex. (**C**). Properties of Gold nanoparticles and calorimetry. (**D**) A generalized illustration on working principle of colorimetric aptasensor when bio-recognition element (BRE) combines with signal transduction element (STE) to make a colorimetric aptasensor to detect small molecular targets.

Biosensors circumvent the above-mentioned limitations to ensure a fast in situ analysis [19]. These kinds of detection techniques exhibit high sensitivity, specificity, excellent performance and are easy-to-use or inexpensive [20]. Biosensors are desirable for rapid or accurate detection of environmental pollutants, materials, biological and chemical stuff [21]. A biosensor is a device involving two main parts in close proximity: a bio-recognition element (BRE) and a signal transduction element (STE) (Figure 1A). BRE comprises two affinity-pairing partners, e.g., receptor/specific ligand, enzyme/substrate and antibody/antigen, etc. One of the partners is usually immobilized [18]. BRE is an essential component of a biosensor. Most common BREs include aptamers, enzymes, molecularly imprinted polymers, antibodies, and many more. These BREs are known to have high specificity and selectivity for their specific targets [20]. The STE is used to detect any interactions between the two affinity-pairing partners and thus converts the biological signals into useful electrical signals [18]. Finally, detection system monitors these signals for further analysis [20]. Biosensors can be grouped or classified based on their STEs and BREs [18]. Based on the choice of BREs, biosensors are classified as aptasensors, MIP-based biosensors, and immune-sensors [20] (Figure 1A).

Biosensors still have their own drawbacks mostly around sterilization, cost and stability. Main disadvantage of these sensors is the uncertainty of biological recognition/sensing element which may affect the overall sensing mechanism by environmental factors (temperature, ionic strength and pH), duration of use and type of molecules. They may have problems associated with transduction element size [18].

## 3. Aptasensors and Aptamers

A kind of biosensor using aptamers as BRE is known as aptasensor [20]. Aptamers are synthetic single-stranded oligonucleotide sequences (RNA or DNA) with high specificity and affinity to bind a variety of target classes including proteins, peptides, drugs, small molecules, whole cells, inorganic and organic molecules, etc. [22]. Aptamers are especially screened via an in vitro selection process named SELEX (systematic evolution of ligands by exponential enrichment) [23], in situ SELEX [24], in-silico/computational approaches [25] and some bioinformatic methods to combine both the in silico and in vitro approaches, etc., to identify the best fit for each target analyte [26]. They have several other striking features such as high stability, greater affinity, low molecular weight, easy and reproducible preparation [27]. They undergo a folding process to make a specific three-dimensional structural rearrangement in order to bind with their particular target (Figure 1B). Three-dimensional structure depends on aptamer sequence, primary structure, pH, temperature and buffer composition of the sensor [28]. Target-aptamer interactions mainly depend on the nature of its binding analyte, three-dimensional structure of the aptamer and distribution of their charges [29]. Interaction types between aptamer target complex involve electrostatic interactions, van der Waals forces, shape complementarity, stacking of flat moieties and hydrogen bonding so that their dissociation constants (*K_d_*) values range from pico- to nanomolar levels [30].

Aptamers can also be referred to as “chemical antibodies” since they mimic antibodies in their applications and characters. Although aptamers are advantageous over antibodies in many aspects especially because of their high affinity, strong specificity, low immunogenicity, targeting a broad range of analytes, cost effectiveness and short preparation cycle [31] they are not as commonly used as antibodies, however developing rapidly [32]. Performance of different aptasensors can be compared keeping in view some important characteristics or parameters of their constituent aptamers such as affinity, specificity, selectivity, limit of detection (LOD), and stability, etc.

## 4. Important Parameters to Measure the Efficiency of Aptasensors

The word affinity stands for the strength of interactions existing between the target and its specific aptamer (of an aptasensor), is measured by approaching the association or binding constant (*K_a_*) that is inversely proportional to the dissociation constant (*K_d_*). Aptamer sequences with lowest K_d_ values have been considered to show strong target interactions. Hence, high-affinity aptamers may bind to even small quantities of target molecules present in the sample (make them more sensitive) known to have a low limit of detection [33]. The selective aptamer binding to only its desired analyte without any cross-reactivity to some non-targeted analytes from an entire sample mixture is termed as the specificity which is a crucial parameter to diminish false-positive results [34]. However, aptamers could not be absolutely specific (in an aptasensor), they can be selective to bind their desired target analytes in the best way and many folds lesser than non-targeted analytes. Because attaining 100% specificity might not be promising, efforts are being made to produce aptamers as selective as possible [33]. The LOD is referred to be the minimum detectable quantity of an analyte in comparison to a blank sample (in the absence of that particular analyte) [35]. The key objective in constructing an efficient biosensor is to approach the highest sensitivity with a smaller LOD than the minimum residual level [36]. The stability of an aptasensor is the capacity to conserve its efficient performance under prevalent situations for a time being. It can be verified by storing the biosensor in a set of specified conditions for a couple of weeks and then relating the analytical efficiency before and after storage [19].

## 5. Classification of Aptasensors

In addition to the built-in biosensor advantages, apta-sensors take another advantage of reusability over the conventional antibodies [37]. Aptamer based biosensors depending on STE have been classified as optical, mass-sensitive, micromechanical and electrochemical sensors [38]. Optical assays have been extensively developed for their simple operation, quick response and high sensitivity. Combining aptamer as BRE with optical bioassays as STE, offer unique advantages to optical aptasensors [39,40]. This kind of aptasensors can be grouped on the basis of their light absorption properties (when they are exposed to different analytes) and luminescence changes [41]. Optical aptasensors are mainly classified into four types including colorimetry, fluorescence, surface-enhanced Raman scattering (SERS) and surface plasmon resonance (SPR) [40] (Figure 1A). In this review, we will focus on AuNPs-based colorimetric aptasensors only.

## 6. Gold Nanoparticles (AuNPs) Based Colorimetric Aptasensors

Nanomaterials are widely used for their excellent sensing application in biosensing with favorably lowered detection limits and higher sensitives. Almost all nanomaterials take the advantage of high specific surface allowing the immobilization of an enhanced amount of bio-recognition element (BRE) to substitute the classic transduction methods. Covalent linkage of nanomaterials to biomolecules is attributed towards the lowering of unspecific physisorption, stability of the system and the reproducibility of the surface functionalization. A variety of nanomaterials, each with their own unique properties are known to enhance biosensor performance [42].

The combination of aptamers with AuNPs has been widely used to develop colorimetric aptasensors for optical recognition of antibiotics [43]. AuNPs have attracted greater attention owing to their light-scattering properties, strong optical absorption, unique chemical, biological, electronic properties, low or no toxicity and fluorescence quenching, etc. [44,45] (Figure 1C). Their unique optical properties are the result of mutual electronic oscillations at their surface which can be controlled by regulating their sharpness, composition and size (diameter) especially. Their diameter can be easily controlled by various experimental parameters to shape-up the desired optical features [46]. Fabricating colorimetric aptasensors by the use of AuNPs is gradually getting popularity for their simple operation and easy preparation [47]. The STE is an exceedingly essential part of an aptasensor while designing colorimetric assays with AuNPs because of their special optical properties, e.g., high extinction coefficient and surface plasmon resonance (SPR) [48]. The aptamers are adsorbed on AuNPs surface (in the absence of their high-affinity target) to stabilize them, thus preventing their salt-induced aggregation because of SPR connection among adjoining particles [49]. Contrarily, on target addition to the aptasensor, aptamer binds to their high affinity target, causing aptamer desorption from AuNPs surface, thus aggregating the AuNPs leading to a color change from bright red to blue or purple (Figure 1D) most commonly (the absorbance peak shifts from 520 to 650 nm) because their unique localized surface plasmon resonance (LSPR) is changed. These absorbance peak shifts could be identified by collecting them on reflectance signals [50] and then be quantified by using a UV–Vis spectrophotometer [49]. As their color changes are very obvious, it can be easily detected by the naked eye. This kind of aptasensors is more appropriate for their in-situ applications where they can greatly facilitate simple and rapid routine inspections [51]. In this review, we have summarized the performance dependent analysis of almost all of the colorimetric gold nanoparticles-based aptasensors reported till now with a special focus on the detection of antibiotics. Colorimetric aptasensors reported for different antibiotics have been outlined individually with respect to the particular group they belong.

AuNPs diameter is the most important factor in all the reported methods with most of the protocols using the particle size between 10 and 27 nm diameter (most of the methods used 13 or 15 nm) of AuNPs, prepared by classical citrate reduction of HAucl_4_ or their modifications for example the method used by Huang et al. [52] to prepare AuNPs. Antibiotics have been classified into seven groups on the bases of their functions and molecular structure, including β-lactams (BLCs), aminoglycosides (AGSs), anthracyclines (ACs), chloramphenicol (CAP), fluoroquinolones (FQs), tetracycline (TC) and sulfonamides (SAs) Table 1.

### 6.1. β-Lactams and Their Residue Detection

β-lactams are a class of antibiotics, which includes cephalosporins, penicillin, carbapenem and monobactams, and represents an effective medicine widely used in the treatment of mastitis courses, pulmonary, urinary and septicemia conditions [53]. They also find uses in boosting animal growth [54]. They are characterized by their representative β-lactam ring [19] (Table 1). An over-use of these substances results in their residues to be accumulated in food, surface water, soil, etc., predominantly in animal-derived foods and other dairy products [55,56]. It is extremely important to detect the traces of these contaminants in all kinds of foodstuffs as otherwise they may pose severe health concerns for human beings, ranging from resistant bacterial evolution to allergic reactions in individuals [57] and bacterial resistance related infections [58]. The most important details of the AuNPs based colorimetric aptasensors being discussed for the antibiotics of each group individually, including the aptamer sequences, dissociation constant (KD), the limit of detection (LOD), color change, AuNP particle size, and synthesis protocol are listed in their respective tables for each antibiotic class (Table 2). 

A penicillin-like antibiotic, ampicillin (AMP) (Table 1) is usually used to treat different bacterial infections, e.g., bronchitis, pneumonia, ear, urinary tract, lung and skin infections [93]. AMP is extensively used in agriculture and medicine for the treatment of bacterial infections and is a member of penam class of beta-lactams. An overdose of AMP could cause undesirable accumulation of its residues in foodstuffs and may lead to serious complications, such as breathing difficulties, seizures, and allergic reactions in humans [94,95]. The first aptamer-based sensor for AMP dual detection (fluorescence–colorimetric) was reported by Song et al. based on AuNPs and ssDNA (selected by magnetic bead-based SELEX). AuNPs acted as a signal probe in developing color for colorimetric assay and as a quencher using fluorescence in a dual-detection system at the same time [59].

In another method, Shayesteh and Ghavami designed a comparative study to investigate the adsorption efficiency of two different aptamers (T-Apt and polyA Apt) on the surface of AuNPs for AMP detection. Their findings suggested that polyA Apt could be a better substitute for thiolated-Apt in the construction of aptasensor to detect a number of analytes including AMP [60].

### 6.2. Aminoglycosides and Their Residue Detection

Aminoglycosides represents an antibiotics class, including kanamycin, tobramycin, streptomycin and neomycin, etc., derived from several Streptomyces species [96]. All of these members share a common hexose ring in their structure, normally streptidine for streptomycin molecule or 2-deoxystreptamine and numerous glycosidically linked amino sugars [97]. Aminoglycosides are known to have broad-spectrum activity, they are mostly used as veterinary drugs to cure infections by aerobic Gram-negative bacteria and many other bacteria. The detection of traces of these drugs in nutrition is considered a big health hazard for the consumers [98].

#### 6.2.1. Kanamycin (KAN)

Kanamycin (Table 1) is an important subclass of aminoglycosides being used widely for the treatment of serious bacterial infections (Gram-negative and Gram-positive) by protein synthesis interference. It shows a narrow range of therapeutic index. An overuse of this medicine in animal-derived nutrients may have serious antibiotic resistance, nephrotoxicity, and ototoxicity in humans [40]. Thus, in order to eradicate these harmful effects, selective, accurate, sensitive and simple detection methods for kanamycin monitoring in animal-derived foodstuff and serum are direly needed [99]. Since selective or sensitive KAN residues detection methods for clinical diagnosis and food safety are highly demanding, more reports have been found that are related to KAN aptasensors than other antibiotics.

**Figure 2:**
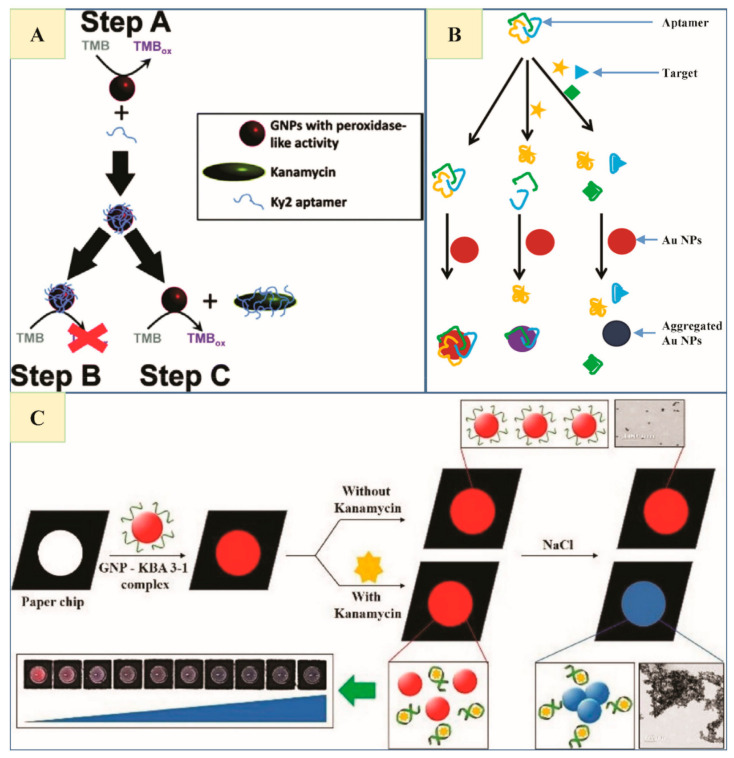
(**A**) Schematic depiction of the ‘turn-on/turn-off’ nanozyme catalytic activity of aptamer-functionalized AuNPs for Kanamycin (KAN) detection (Step A) showing intrinsic peroxidase activity of AuNPs when they ‘turn-off’ after functionalization with Ky2 in the absence of KAN (Step B), and ‘turn-on’ again in the presence of KAN (Step C) (Reproduced with permission from [61] © 2014 Royal Society of Chemistry). (**B**) A schematic illustration of multiplex antibiotic detection using a colorimetric aptasensor based on a simple working principle (Reproduced with permission from [63] © 2014 Plos ne). (**C**) A schematic demonstration of a paper chip-based AuNPs colorimetric aptasensor for KAN detection (Reproduced with permission from [64] © 2017 Elsevier).

A highly sensitive and ultrafast ‘turn-on/turn-off’ method was approached by Sharma et al. that combines Ky2 (ssDNA) aptamer with tyrosine-reduced AuNPs as a peroxidase nanozyme to detect KAN. Nanozyme activity of AuNPs eliminates the necessity for NaCl induced aggregation bringing about faster readouts and improved sensitivity. Overall, a one-step, straightforward, and simple method was developed to take place in 3–8 min [61]. The working principle is shown in Figure 2A.

Song et al. reported a high-affinity ssDNA aptamer for KAN by SELEX (using affinity chromatography). Thus, they constructed a biosensor based on salt induced aggregation of unmodified AuNPs upon aptamer dissociation in the presence of KAN which binds to the loop region, especially the GG region, of the Ky2 aptamer. This low-cost sensor is used in food safety and pharmaceutical preparations [62].

A multiplex colorimetric aptasensor by adsorbing more aptamers (from more than one class, mixed at a particular ratio) on AuNPs surface was designed by Niu et al. to detect more than one antibiotic simultaneously (e.g., KAN, SDM and ADE). In the presence of any one or more targets, the corresponding aptamer undergoes conformational changes to dissociate from AuNP surface- to make aptamer-target complex. AuNPs undergoe aggregation, leading to specific color changes [63] (Figure 2B). The multiplex aptamer-based sensor described above has its own limitations; for example, it fails to determine which analytes were detected from a multiplex sample showing a positive result (Table 3). However, in various situations, e.g., veterinary drug traces determination in animal production, a great deal of different samples need screening for more than one analyte simultaneously. Their multiplex aptasensor working principle can be extended successfully for various other analytes detection by changing the specified aptamer sequences for the desired targets. 

A label-free, nitrocellulose membrane paper chip-based, simple method to visualize a quick absence/presence analysis of chemical reactions by the naked eye, was reported by Ha et al. However, a quantitative analysis can be performed using color and image analysis software. In the presence of KAN, kanamycin binding aptamer is detached from AuNPs surface (to make aptamer-target complex) to induce AuNPs aggregation and bring about the color changes in solution Figure 2C [64].

**Figure 3:**
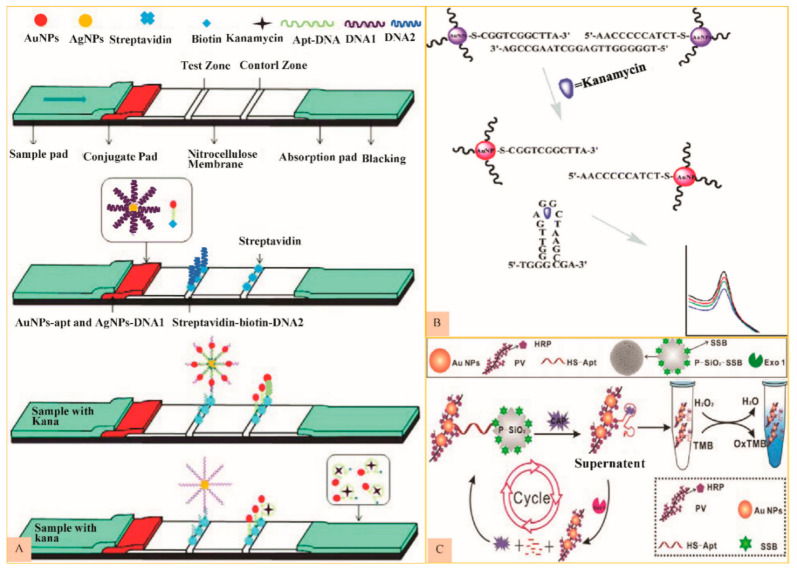
(**A**) Schematic figure showing system alignment and detection principle of strip aptasensor for KAN (Reproduced with permission from [65] © 2018 Royal Society of Chemistry). (**B**) Schematic diagram to represent spectrophotometric kanamycin detection (Reproduced with permission from [66] © 2014 Royal Society of Chemistry). (**C**) Scheme depicting the suggested biosensing of STR dependent on the developed colorimetric aptasensor (Reproduced with permission from [72] © 2017 Elsevier).

A strip based, lateral flow, on-site KAN detection aptasensor was introduced by Liu et al. in which aptamer modified AuNPs act as a probe (AuNPs-apt) while oligonucleotide modified AgNPs as (AgNP-DNA1) signal amplification element. Another complementary capture probe DNA2 was immobilized on the 3’- terminal of AgNPs-DNA1-apt-AuNP complex on the strip. The working principal of this low cost, highly specific and stable aptasensor is shown in Figure 3A, signifying its potential for in-field KAN testing for food safety applications [65].

Zhou, Zhang et al. developed an AuNPs based spectrophotometric aptasensor for KAN detection in milk. Functionalized AuNPs were aggregated upon the addition of KAN specific aptamer because aptamer forms a hybrid with complimentary strand already attached to AuNPs surface. However, upon KAN addition, it binds competitively to the aptamer, disaggregating the AuNPs and a visible change in absorption spectrum of their solution (Figure 3B) [66].

A novel strip-based, portable and stable colorimetric aptasensor was constructed by Abedalwafa et al. in which a nanofibrous membrane carrier having carboxyl group was fabricated by grafting glutamic acid (GA) into cellulose acetate (CA) (G-CA NFMs). KAM aptamer was then labeled on G-CA NFMs. A complimentary strand (cDNA) (as signal probe) of KAM apt-conjugated AuNPs was hybridized with aptamer on G-CA NFMs. KAM addition can disassemble the signal probe by replacing cDNA to bind with aptamer. The method showed excellent results to detect KAN in milk and drinking water [67].

Shayesteh and Khosroshahi introduced the latest strategy based on AuNPs aggregation controlled by the specific interactions between the cationic polymer (PDDA), KAN presence/absence and anti-kanamycin aptamer. AuNPs are stable with aptamer combined PDDA on their surface. On KAN addition, aptamer dissociated from AuNPs surface to bind KAN. The method is used to detect KAN concentration in serum clinically [68].

#### 6.2.2. Tobramycin (TOB)

Tobramycin (Table 1) is an aminoglycoside antibiotic, with broad-spectrum activity, derived from *Streptomyces tenebrarius* [97]. TOB is a cheap drug, widely used in animal farms for animal husbandry leading to its residue accumulation in food chain from animal derived food, such eggs, meat and milk, etc. [49]. Because of its adverse effects such as nephrotoxicity and ototoxicity, it shows a narrow therapeutic range in blood serum same as other aminoglycosides. Monitoring of blood serum levels before therapy is of critical importance for these reasons [97].

Han et al. screened ssDNA aptamers through magnetic bead-based SELEX which they truncated and used to develop AuNP-based photometric aptasensor. Theoretical modeling exhibited that nucleotides 14–18 and 26–29 play a considerable role in aptamer and tobramycin interactions. A truncated 34 nucleotides aptamer was finally optimized in an aptasensor which has promising results in TOB detection in honey samples [69].

TOB detection was designed by Ma et al. based on ssDNA and unmodified AuNPs. The high specificity and sensitivity aptasensors work well on salt induced AuNPs aggregation controlled by aptamer adsorption or desorption on AuNPs surface in the presence or absence of TOB. The suggested system was used successfully to detect TOB in chicken eggs and raw milk [49].

#### 6.2.3. Streptomycin (STR)

Streptomycin (Table 1) belongs to the aminoglycoside antibiotic group that is used widely in the veterinary and medical practices to cure infections caused by gram-negative bacteria [100]. Higher accumulation of STR residues results in evolution of pathogenic resistant strains on human tissues after ingestion of infected foodstuffs, which becomes a big risk to livestock and human health [101].

Zhou et al. screened and identified streptomycin aptamer STR1 by affinity magnetic beads-based SELEX which they used to construct an AuNPs-based colorimetric sensor. Competitive binding of STR with its aptamer brings out AuNPs aggregation in salt solution and a visible color change. The sensor showed high affinity and specificity for STR detection in honey samples [70].

Liu et al. isolated a high affinity A15 aptamer to detect STR after 10 SELEX rounds (via affinity chromatograph). Aptamer sequences were used to construct aptasensor based on AuNPs aggregation method. The technique is used to detect antibiotics and other small molecules in food industries [71].

A point of care testing (POCT) STR detection sensor using AuNPs labelled PV (as Nano tracers) and SSB labeled porous silica (as capture probes) and exonuclease-assisted target recycling (for signal amplification) was developed by Luan et al. The system depends on the higher affinity of aptamer towards STR than SSB (single stranded DNA binding protein). When exonuclease I and STR are added, the nano tracer combines with STR to form STR/Apt-Au-PV complex. After this, Exo I keep on digesting the aptamer attached to the complex, and STR is released again to take part in a new cycle. The sensor generates more nano tracers into the solution to boost the sensitivity (Figure 3C) [72].

Zhao et al. reported peroxidase-mimicking activity of AuNPs and their behavior towards the STR-apt complex. AuNPs might oxidize peroxidase substrate in the presence of H_2_O_2_. The system is easy to fabricate for large-scale applications. It is rapid, accurate, and can be used to detect a wide range of other small molecules by replacing their particular aptamer sequences [73].

A colorimetric and fluorescence quenching STR detection aptasensor based on double-stranded DNA (dsDNA) and aqueous AuNPs was designed by Emrani et al. In samples lacking STR, aptamer/FAM-labeled CS forms a stable dsDNA, leaving behind salt aggregated AuNPs with a color change in the solution. Contrarily, aptamer binds to STR, while CS stabilizes the AuNP hence no color change. Sensors worked well in serum and milk samples [74].

#### 6.2.4. Neomycin B (NB)

Neomycin (Table 1) is an aminoglycoside, derived from *streptomyces fradiae*, partially active against Gram-positive bacteria and shows excellent results for Gram-negative bacteria. NB is being widely used in the recent decade to cure gastrointestinal infections of poultry, goats, pigs, sheep, cattle and through intramammary administration to cure mastitis [102]. However, an overdose of neomycin is potentially well-known for its nephrotoxic and ototoxic effects in animals and humans [103]. Thus, it is essential to introduce sufficiently sensitive strategies for neomycin residue detection animal-derived foodstuffs. Unfortunately, only one aptasensor has been reported for neomycin B detection so far.

Khavani, Izadyar et al. theoretically designed high affinity RNA aptamers for NB by employing molecular dynamic (MD) simulations which they used to fabricate AuNP based aptasensor to compare the affinity with a wild type aptamer (AP-W). The resulting LOD of AP-M18 was considerably lower than those of other aptamers suggesting its efficiency as a high affinity aptamer for NB detection [75].

### 6.3. Anthracyclines and Their Residue Detection

Anthracycline antibiotics could inhibit DNA replication by inhibition and intercalation of topoisomerases. Clinically, they are used in tumor therapy but their long-term use can have severe side effects such as heart failure [19]. The basic structure, common in all anthracyclines, is shown in green (Table 1).

Daunomycin (Table 1) belongs to the anthracycline group of antibiotics are widely used as cancer chemotherapeutic agents. However, DNR is a major carcinogenic, nephrotoxic and mutagenic itself. It could reach drinking water and may contaminate soil if its residue level is not controlled, eliminated or degraded [104].

He et al. constructed the only sensitive method to be used for on-site food safety against DNR by aptamer based AuNPs aggregation. Since no modification of AuNPs or aptamers is required, the method is low cost, precise and has only 5 min reflection time [76].

### 6.4. Chloramphenicol (CAP) and Their Residue Detection

Chloramphenicol (Table 1) is another broad-spectrum group of antibiotics, [105] widely used in aquaculture and animal husbandry for their efficiency in inhibiting bacterial growth. However, CAP brings severe side effects on the human hematopoietic and digestive system once their residues are ingested through the nutrition [106]. The excessive accumulation of CAP residues in human blood and animal-derived foodstuffs pose adverse effects on humans, such as gray baby syndrome, aplastic anemia, and leukemia [77].

**Figure 4:**
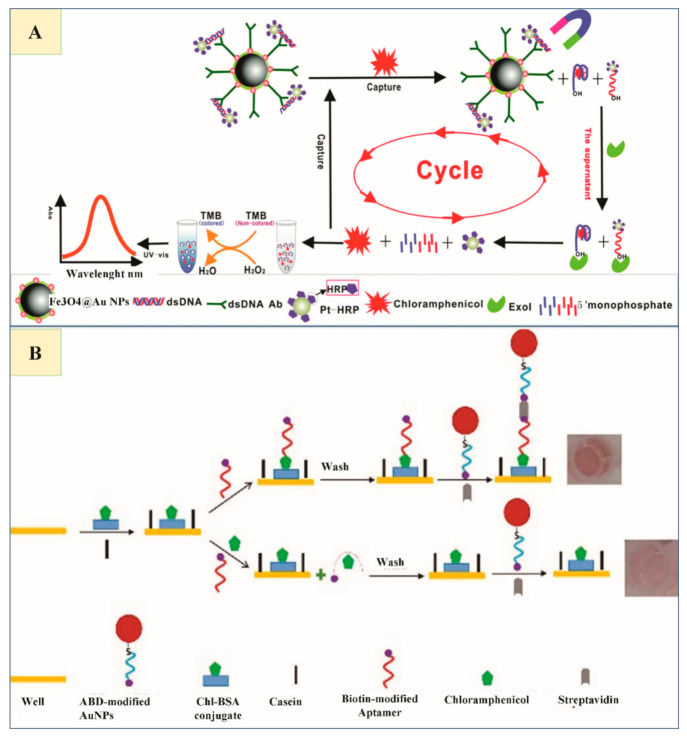
(**A**) Illustration of the detection processes related to color change based on chloramphenicol (CAP) presence or absence (Reproduced with permission from [78] © 2015 Royal Society of Chemistry). (**B**) Schematics showing CAP detection based on AuNPs colorimetric method. In the absence of target, the sandwich aptasensor results in bright red color. In the presence of CAP, modified AuNPs cannot bind to the well, with a subsequent pale red color change (Reproduced with permission from [77] © 2016 Elsevier).

A sensitive, facile and selective method for ultra-trace level residue detection of CAP was devised by Miao et al. which can be efficiently used for the detection of other antibiotics as well by changing the aptamer sequences. Sensor relies on triple signal amplification approach with an Exo I-assisted target recycling and magnetic aptamer-HRP co-immobilized PtNPs nano tracers. This biosensor preparation is complicated (Figure 4A) [78].

Abnous et al. introduced a sandwich aptasensor for CAP detection, based on an indirect competitive enzyme-free setup using biotin, AuNPs and streptavidin. The sandwich structure forms in aptasensor when the sample lacks CAP residues, displaying a sharp red color. On CAP addition, functionalized AuNPs fail to bind 96-well plates, characterized by a faint red color (Figure 4B) [77].

**Figure 5:**
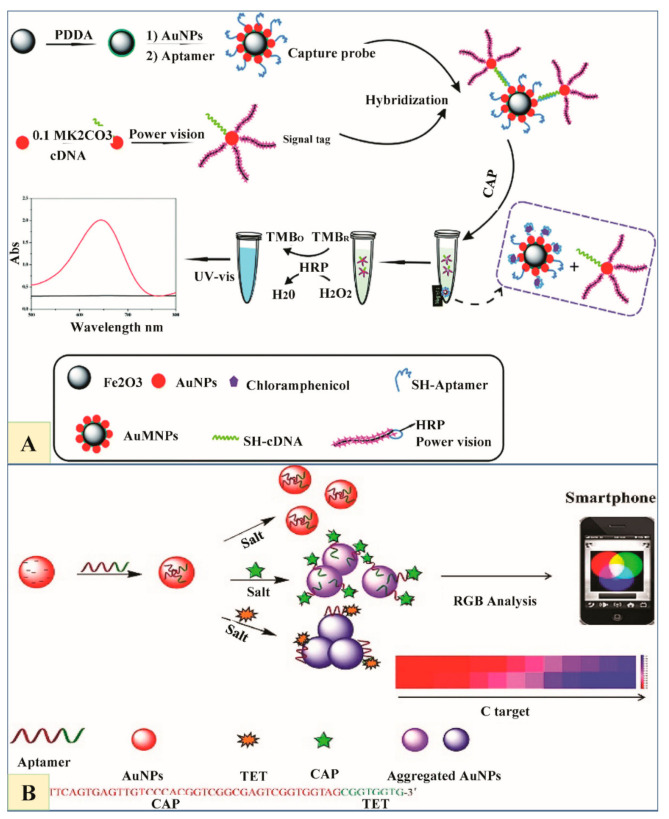
(**A**) Scheme showing the proposed working principle of CAP detection using cDNA–AuNPs–PV as a signal tag (Reproduced with permission from [79] © 2015 Royal Society of Chemistry). (**B**) Schematic diagram exhibiting TET/CAP detection in which the multi-Aptamer acts as a molecular switch adjusting the AuNPs aggregation. When a specific target removes the fragment of its particular Apt from the AuNPs surface, unbalanced AuNPs aggregation occurs at different scales under high-salt conditions, causing colloidal color changes, which can be detected by UV-spectrum and Smartphone analysis, respectively (Reproduced with permission from [81] © 2020 Elsevier).

PV labeled AuNPs were modified by conjugation with cDNA (cDNA–AuNPs–PV) as signal amplifiers for CAP detection by Gao et al. They then functionalized a CAP aptamer by immobilizing it on Fe_3_O_4_@Au magnetic NPs (AuMNPs–Apt) as capture probe. Special tags might hybridize with cDNA and aptamer to form AuMNP– Apt/cDNA–AuNP–PV conjugates. When CAP is added, it binds to aptamer, dissociating some cDNA–AuNPs–PV on the conjugates with magnetic separation. At this point, PV is catalyzing 3,3’5,5’-tetramethylbenzidine (TMB), which brings out a color change that can be quantified by (UV-vis) spectroscopy (Figure 5A) [79].

An aptasensor for CAP ultra-trace residue detection based on the use of a new enzyme-polymer nano tracer (as a signal amplifier) was developed by Miao et al. HRP-Au composite was labelled by enzyme-linked polymer (EV) which could efficiently catalyze TMB oxidation via H_2_O_2_, causing visible color changes. This simple and sensitive assay find excellent applications in food industries and in situ food safety especially in fish samples [107].

Huang et al. combined aptamer and peroxidase-mimicking DNAzyme-functionalized AuNP nanoprobe for sensitive and rapid CAP detection. They prepared nanoprobe by the sulfhydrylation assembly of cDNA against CAP aptamer and high content hemin/G-quadruplex DNAzyme on the surface of AuNPs. Because of the high efficiency of the nanoprobe signal amplification, the sensitivity of this method rivals several electrochemical biosensors [80].

Javidi et al. developed a CAP detection method in milk using intact long-oligonucleotide aptamers with aptamer terminal locks (ATL). In the ATL approach, long aptamers were used as molecular recognition probes while short-sequences worked as locker probes (LP) which must be complementary to aptamer terminal fragments. The method relies on the interaction of ssDNA (LP) with AuNPs, target-induced release of LP from long aptamer and no interaction of ATL with AuNPs [82].

A biosensor for CAP detection method, in which ssDNA-functionalized AuNPs aggregation was supported by lanthanum (La^3+^) ions and smartphone imaging was described by Wu et al. However, La^3+^ as a trigger agent might limit the on-site application because of poor biocompatibility and storage instability. Moreover, the sensitivity and specificity of the analytical performance needs improvement for future studies [51] (Table 3). 

Wu, Huang and Wu later devised another protocol for multiplex antibiotics (CAP and TET) detection, based on multifunctional ss-DNA apt coordinately controlling AuNPs aggregation. When one analyte is added into the solution separately, that specific recognized sequence of aptamer binds to it and is dissociated from AuNPs, whereas the non-specific, unrecognized part coordinately controls the salt-induced aggregation of AuNPs [81] (Figure 5B).

### 6.5. Fluoroquinolones (FQs) and Their Residue Detection

Fluoroquinolones belong to a synthetic group of antibiotics, originated from quinolone nalidixic acid by the addition of a fluorine atom at carbon 6 and piperazine at carbon 7 position [108]. FQs kill bacteria or inhibit their growth by inhibiting their DNA replication favored by their chemical structure [109] (Table 1). Since the invention of FQs in the 1970s, and their use in livestock and human therapeutics in the 1990s, they became commonly-prescribed antibiotics for their broad-spectrum mode of action towards Gram-negative, Gram-positive bacteria and mycoplasma [110]. However, due to their widespread use, they can easily enter the environmental water table by human defecation into the sewage system or spreading of compost onto the agricultural lands, which may cause food-borne bacterial emergence in humans or severe allergic reactions [111].

Ciprofloxacin belongs to FQs, used to cure various bacterial infections such as respiratory infections, gastrointestinal diseases and urinary tract infections. Being a broad-spectrum antibiotic, CIP is highly prescribed to be used for livestock and humans [112]. It can enter the drinking water and environment in a number of ways, including pharmaceutical production emissions, domestic sewage, solid waste disposal, landfill dumping, and livestock manure, etc. [113]. CIP has been detected at a considerably higher concentration in river waters [114].

Lavaee et al. constructed the only CIP detection assay based on modified AuNPs with cDNA and CIP aptamer. Flower-shaped modified AuNPs surface show enzyme-like activity which catalyzes nitrophenol reduction by NaBH_4_. The method completes in one hour and is used broadly to detect CIP in real samples such as milk, water, and serum [83].

### 6.6. Tetracyclines and Their Residue Detection

Tetracyclines are a broad-spectrum group of compounds employed commonly because of their low cost in comparison to other antibiotics. At present, more than 20 different tetracyclines are available on the market; however, oxytetracycline, doxycycline, tetracycline, and chlortetracycline are among broadly used in veterinary medicine [115]. Their name is derived from their basic molecular structure (Table 1) comprised of six linearly arranged rings [19]. Excessive use of TET as antibiotics and a growth promoter in veterinary medicine is causing its residue accumulation in foodstuffs, which is a grave risk for human health and the environment [116].

#### 6.6.1. Oxytetracyclines (OTC).

OTCs are the major antibacterial agents, inhibiting the bacterial protein synthesis by binding to 30 S ribosomal subunit reversibly. However, the overuse of these drugs is causing serious problems because of their residue accumulation in animal derived foods, especially milk, which is directly toxic or can cause severe allergic reactions to many hypersensitive individuals [117].

Kim et al. employed a high specificity OTC binding ssDNA aptamer that can induce unmodified AuNPs aggregation by desorption from their surface resulting from the target-aptamer interactions. The protocol can be optimized for other unmodified AuNPs-based aptasensors [84].

**Figure 6:**
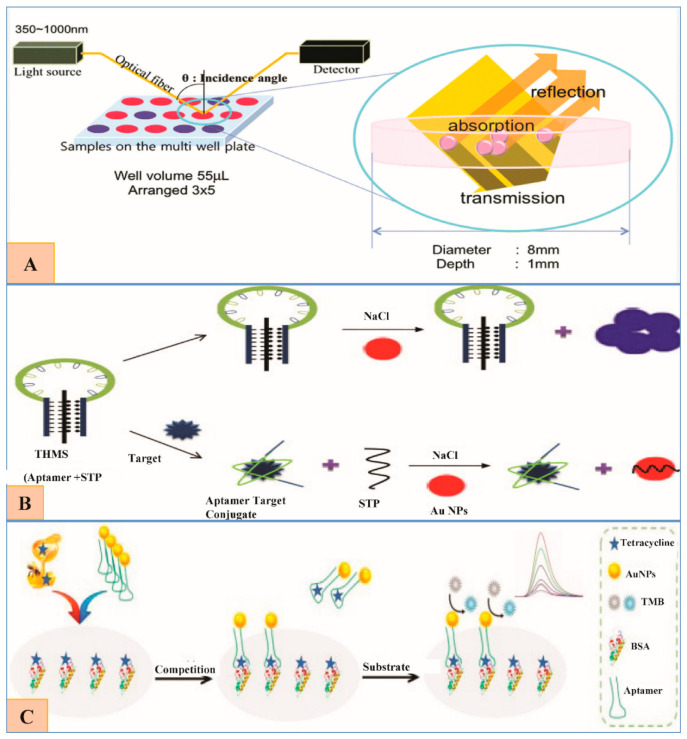
(**A**) The schematics showing the reflectance-based multi-well plate colorimetric aptasensor using AuNPs (Reproduced with permission from [50] © 2015 Royal Society of Chemistry). (**B**) Schematic explanation of TET detection based on colorimetric triple-helix molecular switch (THMS). In the absence of Tetracycline (TET), THMS (Aptamer + STP) remains stable, resulting in AuNPs aggregation by salt, color changes from red to blue. In the presence of TET, aptamer binds to its target, the signal transduction probe (STP) leaves the THMS and adsorbs on the surface of AuNPs thus stabilizing them, so no color change (Reproduced with permission from [87] © 2015 Elsevier). (**C**). Illustration of the proposed gold nanoparticle-linked competition-based aptamer assay (Reproduced with permission from [89] © 2020 MDPI).

A reflectance-based aptesensor that operates at two wavelengths (520 and 650 nm) for OTC detection was devised by Seo et al. They found that their sensor generates more sensitive and stable signals as compared to the absorbance-based assays based on AuNPs aggregation even at higher concentrations (Figure 6A). They considered it a better approach for the portable detection of such small molecules by using aptamers [50].

A low cost, rapid, high selectivity and convenient DNA aptasensor for KAN and OTC was constructed by Xu et al. After the occurrence of recognition events, DNA sequences released from MB surface and DNA probes captured HRP modified AuNPs. Consequently, o-phenylenediamine and 3,3′,5,5′-tetramethylbenzidine were oxidized by horseradish peroxidase displayed color changes [85].

Kazerooni et al. designed an OTC aptasensor based on salt-induced AuNPs aggregation by OTC aptamer interaction in the presence or absence of target. The sensor is used in the field of food safety and biomedicine as a promising tool and can be extended for other antibiotics by replacing aptamer sequences [5].

#### 6.6.2. Tetracycline (TET)

The TET misuse in aquaculture and animal husbandry results in the accumulation of its residues at higher levels in environment, such as water and soil [118]. Owing to its life-threatening effects, it is very important to detect the trace levels in an effective and efficient manner (Table 1 for TET structure).

He et al. proposed a TET detection strategy based on the aggregation of AuNPs, controlled by TET aptamer interaction with TET and hexadecyltrimethylammonium bromide (CTAB). CTAB is a surfactant to control the shape and size of AuNPs and form a complex with aptamer in the absence of target, thus aggregating AuNPs and bringing out a significant color change [86].

He et al. introduced a simple TET detection method based on the AuNPs aggregation controlled by TET, aptamer and poly diallyldimethylammonium (PDDA). Cationic polymer PDDA controls AuNPs aggregation and aptamer hybridization. TET addition reduces aptamers to form an aptamer-TET complex; thus, cationic polymers aggregate AuNPs and bring out a significant color change [119].

A fast, selective and sensitive TET detection (in blood serum and milk) aptasensor was designed by Ramezani et al. based on AuNPs and triple-helix molecular switch (THMS). THMS exhibits numerous advantages including preserving high selectivity, original aptamer affinity, stability, and sensitivity. THMS system commonly comprises a label-free target-specific aptamer, a dual-labeled oligonucleotide as a signal transduction probe (STP) and two arm segments (Figure 6B) [87].

A specific and sensitive TET detection system was documented by Luo et al. using negatively-charged TET aptamer and cysteamine-stabilized AuNPs (CS-AuNPs) probe which is positively-charged. The method is particularly useful for real-time and on-site TET detection in milk [88].

In a recent study by Sheng, Liang et al., TET-BSA were coupled by glutaraldehyde crosslinking assay and applied to the microplate for competitive binding (of free TET in sample and fixed TET in microplate) with aptamer. Concurrently, TET aptamer-labeled AuNPs (TET apt-AuNPs) were prepared. In this way, a novel competitive TET detection sensor for honey was developed based on greater catalysis ability of AuNPs and higher aptamer selectivity (Figure 6C) [89].

### 6.7. Sulfonamides (SAs) and Their Residue Detection

SAs (Table 1) are one of the main antibiotic groups that are extensively used in human medicine, aquaculture, and livestock husbandry. SAs are recently detected far and wide in the aquatic ecosystem, posing health risks to living organisms [120]. SA residues in foodstuffs envisioned for human ingestion is a world-wide apprehension since they are evolving antibiotic resistance, allergic for humans and carcinogenic [121]. The hydrogen atom on sulfamine is usually substituted by a variety of heterocyclic rings to make different kinds of sulfonamides [122].

#### Sulfadimethoxine (SDM)

SDM (Table 1), is a major class of livestock and veterinary importance antibiotics, used widely for the management of coccidiosis [123]. It is used as feed additive for the prevention and treatment of various animal diseases because of its broad-spectrum antibacterial activity. However, the misuse of SDM is associated to severe health hazards; for example, acute haemolytic anemia [124]. Consequently, concerns for SDM residue detection in animal-derived food, especially milk, honey, eggs, meat and other foodstuffs, have been raised.

A fast, label-free and simple SDM detection sensor was optimized by Chen et al. based on controlling the aptamers to AuNPs relative amount, ionic strength by salt addition and the pH value of AuNPs solution [90].

Yan et al. reported a reliable, cost-effective and sensitive colorimetric sensor for SDM detection relies on peroxidase-like catalytic activity of AuNPs. SDM-apt undergoes desorption from AuNPs surface in the presence of SDM which reactivates the catalytic activity of AuNPs. Color change is dependent on SDM concentration and can be seen by UV-visible absorptiometry at 650 nm [91].

Chen at al. recently devised a label-free, dichromatic sensor for SDM detection. Dichromatic mode was achieved by AuNPs color changing and fluorescence at 530 nm. SDM-apt dissociate from the AuNP surface in the presence of SDM to make an aptamer target complex which shows color changes from in the solution and fluorescence by adding syber green I and cDNA [92].

## 7. A Generalized Overview, Future Perspective, Challenges and Conclusions

Because the development of innovative or emerging methods for accurate antibiotic detection is an environmental and clinical need, we gave a comprehensive overview of the reported aptasensor advances over the past decade. Some of the aforementioned aptasensors are already being used at commercial level by food industries to detect antibiotic residues for food safety. Some new methods are still in theoretical and early testing phase with a high potential for marketing in the future. Colorimetric aptasensors are highly advantageous as the experimental results can be directly observed/quantified by analyzing visual color changes via naked eye, spectrophotometer or mobile phone chromatism without any kind of complex equipment/instrumentation. Mostly, 5’-end of the different aptamer sequences were used, preferably for immobilization purposes. The specificity of the used aptamer sequences towards their analytes (in many protocols) was verified by testing their sensitivity against possible interfering or structurally similar substances. Nanotechnology made a significant contribution in the field of biosensing; e.g., combining particular aptamer sequences with different nanomaterials led to the development of highly selective and sensitive aptasensors. There are numerous similarities and differences between various sensing protocols as described previously in this review such as, all colorimetric aptasensors discussed here, involves the use of a sensing or recognition material (aptamer) and a colorimetric agent (AuNPs) for signal transduction. From the sensing viewpoint, the inherent properties of AuNPs also offer numerous benefits for the construction of smart sensors, e.g., surface adsorption, dynamic light scattering with the change in their particle size, and the salt-induced aggregation of the AuNPs. However, not all of these aptasensors have the same recognition systems or obey the same working principle (already described for several reports). Subsequently, these kinds of portable devices are simple to operate, rapid, inexpensive, and suitable for on-site detection applications with high sensitivity, selectivity and flexibility. However, some limitations and challenges still need to be addressed here.

All of the protocols used DNA aptamer sequences. RNA sequences should also be characterized for their use in this kind of detection methods. Most of the reported aptasensors are intended to be extended for the detection of a number of different low molecular weight analytes (other antibiotics) by changing the particular aptamer sequence with the most suitable one against the other intended targets to be detected. Unfortunately, not enough high-affinity aptamer sequences have been developed to be replaced for an already existing protocol. We have cited 40 different protocols reported over the decade so far, with most of the studies using the same once-developed aptamer sequence for a specific analyte again and again in different protocols. Despite numerous advances currently made in the field of aptamer based biosensing platform, they are still considered immature if we compare them with immunosensors. This may be attributed to a limited high efficiency aptamer variety available at present. Screening of class specific aptamers to detect common group members can also be helpful to recognize more antibiotic types. Thus, there is an urgent need for more high affinity aptamer sequences to be screened for a wide range of antibiotics (small molecular targets) in the future. Aptamer immobilization approaches, especially their bioconjugation with AuNPs or other nanomaterials, may sometimes confine the aptamer’s target recognition capacity as there is relatively less knowledge regarding the surface-immobilization techniques for aptamers. There is an increasing demand to develop some novel modification and immobilization strategies concerning greater nanomaterials biocompatibility so that the aptamers may attach easily to their functional signal reporters. Because the on-site detection of the antibiotic targets is challenging, it needs to be given more attention. Additionally, environmental or food sample (to be analyzed) conditions, e.g., viscosity, ionic strength, pH, as well as non-specific interactions of the sample matrix with aptamer, must be considered.

Further studies are required to investigate the competing ligand interference, in other words to address the false-negative/ false-positive results or to confirm the selectivity of the aptasensor against their specific target antibiotic. Most importantly, attention is needed to fill the gap of certain parameters (such as pH, temperature, ionic strength, etc.) for the compatibility between aptasensor design and aptamer selection (selection conditions should be same) so as to improve the identification capacity of the aptamer which may indirectly enhance the performance of the sensor in terms of stability, specificity, affinity and sensitivity. Besides this, some external factors may also easily influence the colorimetric assays such as the color of the sample and background fluorescence. Some other challenges are associated with longer fabrication time duration and poor stability or reuse.

By considering the significant advances in the above-mentioned directions, we aim to inspire more efforts for the development of AuNPs based colorimetric aptasensors for on-site detection of antibiotics and other small molecule pollutants. In the end, we would like to recommend improvements by combining various highly efficient AuNPs-based colorimetric aptasensor strategies on one platform to allow multiple residue detections from the only sample as a source. Overall, although there is still a long way to go, we are hopeful that aptamer-based biosensing methods, especially AuNPs calorimetry, will ultimately be proved to be a real-world tool, meeting the present and future challenges which might be impossible otherwise, with the latest available methods. Thus, more efforts are required to manufacture more commercially available aptasensors with high efficiency, easy operation, and low-cost.

Fast and precise assays for the detection of antibiotics still face certain limitations such as sensitivity, specificity, linearity, turnaround time, cost effectiveness, health risks, etc. Nanotechnology is believed to be key to answering these questions while minimizing systemic toxicity. Aptamers offer several advantages over other materials, such as high binding specificity and physicochemical stability, simple modification, long shelf life, minor variation, slight denaturation susceptibility, and cost effectiveness. They interact with a variety of molecules including antibiotics. The enzyme-like activities of gold nanoparticles potentiate their role in biosensing systems and opens the road for their application in various fields of scientific research, diagnostics, and therapy. These superiorities motivate the development of aptasensors for wider applications.

## Figures and Tables

**Table 1:** Classification of seven antibiotic classes (basic structure highlighted in green), and their different derivatives (alterations from green to black molecular structures).

Antibiotic Class	Antibiotic	Molecular Structures
β-Lactams (BLCs)	Ampicillin (AMP)	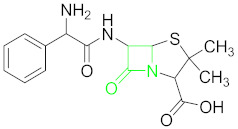
Aminoglycosides (AMGs)	Kanamycin (KAN)	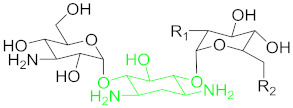
	Tobramycin (TOB)	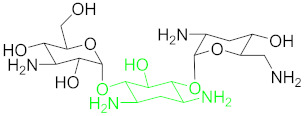
	Streptomycin (STR)	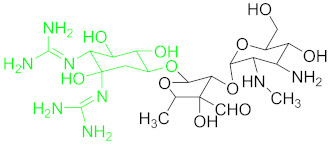
	Neomycin B (NB)	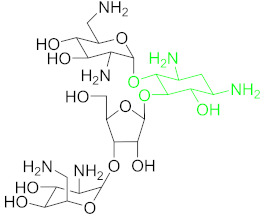
Anthracyclines (ACs)	Daunomycin (DNR)	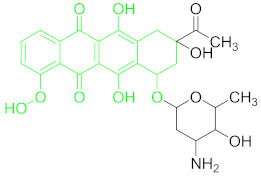
Chloramphenicol	Chloramphenicol (CAP)	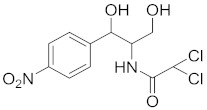
Fluoroquinolones (FQs)	Ciprofloxacin (CIP)	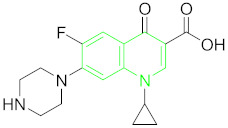
Tetracyclines (TCs)	Oxytetracyclines (OTC)	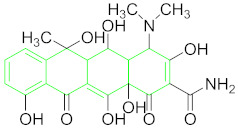
	Tetracycline (TET)	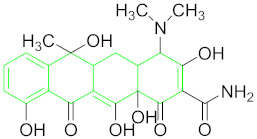
Sulfonamides (SAs)	Sulfadimethoxine (SDM)	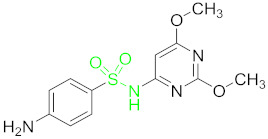

**Table 2:** Summary of important parameters/results for each method discussed (aptamer sequence, linker and spacers, dissociation constant (*K_d_)*, limit of detection (LOD), color change in the presence or absence of target, AuNPs particle diameter and preparation methods for each method, mentioned in the corresponding references (Ref.).

Target	5′ Linker and Spacer	Aptamer Sequences 5′→3′	3′ Linker and Spacer	KD (nM)	LOD (nM)	Color Change	AuNP Particle Size (nm)	AuNP Preparation Method	Ref.
AMP	FAM	AMP17: GCG GGC GGT TGT ATA GCG G AMP18: TTA GTT GGG GTT CAG TTG G AMP4: CAC GGC ATG GTG GGC GTC GTG	biotin	13.4 9.8 9.4	14.3 (dw) 28.6 (m)	Red-purple	13	classical citrate reduction method	[59]
AMP	(SH)–(CH_2_)_6_	-GCGGGCGGTTGTATAGCGG GCGG GCGGTTGTATAGCGG-	(T)_15_–(A)_12_	0.10 0.49	10 50	Red-purple Red-purple	10	Smith method	[60]
KAN	-	TGG GGG TTG AGG CTA AGC CGA	-	8.38	1.49	Ruby red to purplish-blue or colorless	-	classical citrate reduction method	[61]
KAN TOB	-	TGGGGGTTGAGGCTAAGCCGA	-	KAN = 78.8 KAN B = 84.5 TOB = 103	25	Red-purple	13	classical citrate reduction	[62]
KAN SDM	-	SDM; GAGGGCAACGAGTGTTTATAGA KAN: TGGGGGTTGAGGCTAAGCCGA-	-	-	-	Red-purple/blue	13	Classical citrate reduction	[63]
KAN	-	(KBA) 3-1; CGG AAG CGC GCC ACC CCA TCG GCG GGG GCG AAG CTT GCG Ky2; TGG GGG TTG AGG CTA AGC CGA	-	-	3.35 26.8	wine red to purple blue	14, 21, 27	citrate reduction of HAuCl_4_	[64]
KAN	Apt; HS–(CH_2_)_6_ DNA1; SH–(CH_2_)_6_ DNA2; biotin	Apt; TGG GGG TTG AGG CTA AGC CGA DNA 1: TCA GTC GGC TTA GCC GTC CAA CGT CAG ATC C DNA2: CCG ATG GAT CTG ACG T	apt: biotin	-	0.0778	Red- colorless or faint red	13	citrate reduction of HAuCl_4_	[65]
KAN	ssDNA1; HS–(CH_2_)_6_-	KSA; TGGGGGTTGAGGCTAAGCCGA ssDNA1; CGGTCGGCTTA ssDNA2; AACCCCCATCT	ssDNA2; (CH_2_)_3_–SH	-	01	Red-purple	13	citrate reduction of HAuCl_4_	[66]
KAN	KMC apt; NH_2_	KMC apt; TGGGGGTTGAGGCTAAGCCGA cDNA; TCGG CTTAGCCTCAACCCCCA	-	-	2.5	Pink to white	15–20	citrate reduction method	[67]
KAN	-	TGG GGG TTG AGG CTA AGC CGA	(T)_15_–(A)_12_	-	0.05	Red to blue	15	citrate reduction method	[68]
TOB	-	GGG ACT TGG TTT AGG TAA TGA GTC CC	-	-	23.3	red-purple-blue	13	classical citrate reduction	[49]
TOB	-	-	-	-	37.9	red to purple	-	-	[69]
STR	-	I: GGG GTC TGG TGT TCT GCT TTG TTC TGT CGG GTC GT II: TGA AGG GTC GAC TCT AGA GGC AGG TGT TCC TCA GG III: AGC TTG GGT GGG GCC ACG TAG AGG TAT AGC TTG TT IV: TGT GTG TTC GGT GCT GTC GGG TTG TTT CTT GGT TT	-	I: 199.1 II: 221.3 III: 272.0 IV: 340.6	200	red to purple	13	citrate reduction of HAuCl_4_	[70]
STR	I: FAM II: FAM III: FAM	I: CCC GTT TAA AGT AGT TGA GAG TAT TCC GTT TCT TTG TGT C II: GTG CGT TAT AAA CTA GTT TTG ATT CAA TGT TGG GTG TGG G III: GGG CCT GTT TTG CCT TCA CGT TCT CTT CCT TGC CGT TCT G	I: biotin II: biotin III: biotin	I: 6.07 II: 8.56 III: 13.14	25 nm/L	red to purple	15	citrate reduction of HAuCl_4_	[71]
STR	SH–(CH_2_)_6_-	TAG GGA ATT CGT CGA CGG ATC CGG GGT CTG GTG TTC TGC TTT GTT CTG TCG GGT CGT CTG CAG GTC GAC GCA TGC GCC G	-	-	0.0017 (b) 0.001	colorless to blue	19.73	Au NPs–PV	[72]
STR	-	TAGGGAATTCGTCGACGGATCCGGGGTCTGGTGTTCTGCTTTGTTCTGTCGGGTCGTCTGCAGGTCGACGCATGCGCCG	-	19.1	86	red-grayish green	13	citrate reduction of HAuCl_4_	[73]
STR	-	STR apt: TAG GGA ATT CGT CGA CGG ATC CGG GGT CTG GTG TTC TGC TTT GTT CTG TCG GGT CGT CTG CAG GTC GAC GCA TGC GCC G CS; C GGC GCA TGC GTC GAC CTG CAG ACG ACC CGA CAG AAC AAA GCA GAA CAC CAG ACC CCG GAT CCG TCG ACG AAT TCC CTA	-	-	73.1 (b) 102.4 (b) 108.7 (m)	red to blue	15	citrate reduction of HAuCl_4_	[74]
NB	-	AP-W; GGACUGGGCGAGAAGUUUAGUCC Ap-M18; GGACUAAACGAGAAGUUUAGUCC Ap-M20; GGACUAAACGAGAAGCCCAGUCC	-	-	470 27 360	pink red to Blue	13	citrate reduction of HAuCl_4_	[75]
DNR	-	GGGAATTCGAGCTCGGTACCATCTGTGTAAAAGGGGTGGGGGTGGGTACGTCTAGCTGCAGGCATGCAAGCTTGG	-	-	17.1	red to purple	13	reduction of HAuCl_4_	[76]
CAP	AuNPs DNA; Biotin	Apt; ACTTCAGTGAGTTGTCCCACGGTCGGCGAGTCGGTGGTAG AuNPs binding DNA; ACTTTCCATTCCTTTTAC	Apt; Biotin AuNPs DNA; Thiol	-	0.451 (b) 0.697 (m) 0.601	bright red to faint red	14	reduction of HAuCl_4_	[77]
CAP	apt: SH–(CH_2_)_6_ cDNA: SH–(CH_2_)_6_	apt: ACT TCA GTG AGT TGT CCC ACG GTC GGC GAG TCG GTG GTA G cDNA: CTA CCA CCG ACT CGCG CGA CCG TGG GAC AAC TCA CTG AAG T	-	-	0.00093 (b) (0.3 × 10−9 g/L)	colorless to light blue	20	slight modified Frens method	[78]
CAP	apt: SH–(CH_2_)_6_ cDNA: SH–(CH_2_)_6_	apt: ACT TCA GTG AGT TGT CCC ACG GTC GGC GAG TCG GTG GTA G cDNA: TTT TCT ACC ACC GAC TCG C	-	-	0.02 ng/mL	colorless to light blue	20	slight modified Frens method	[79]
CAP	Apt; SH–(CH_2_)_6_ cDNA: SH–(CH_2_)_6_	Apt; ACT TCA GTG AGT TGT CCC ACG GTC GGC GAG TCG GTG GTAG cDNA: CTA CCA CCG ACT CGC CGA CCG TGG 142 GAC AAC TCA CTG AAGT	-	-	0.046 (b) (0.015 × 10−6 g/L)	colorless to light blue	16	slight modified Frens method	[78]
CAP	Apt;Biotin DNAzyme; (SH–(CH_2_)_6_– cDNA; (SH)–(CH_2_)_6_	Apt; ACT TCA GTG AGT TGT CCC ACG GTC GGC GAG TCG GTG GTA G DNAzyme; AAAAAA GGG TAG GGC GGG TTG GG cDNA; AAA AAA AAA AAA AAA AAA AAA AAA AAA AAA CTA CCA CCG ACT CGC C	-	-	0.13 pg/mL	red to blue	13	reduction of HAuCl_4_	[80]
CAP	-	ACTTCAGTGAGTTGTCCCACGGTCGGCGAGTCGGTGGTAG	-	-	7.65 5.88	red to blue	15	Huang et al.	[51]
CAP TET	-	Multi-apt; ACTTCAGTGAGTTGTCCCACGGTCGGCGAGTCGGTGGTAGCGGTGGTG	-	-	CAP.7.0 TET.32.9	(CAP) wine red to purple (TET) wine red to blue	15	Huang et al.	[81]
CAP	-	Apt; ACT TCA GTG AGT TGT CCC ACGGTC GGC GAG TCG GTG GTAG LP; CTGAAGTTCTACCAC	-	-	0.03	pink red to blue	14	Classical reduction of HAuCl4	[82]
FQs CIP	CS2; Thiol	Apt; ATACCAGCTTATTCAATTGCAGGGTATCTGAGGCTGATCTACAATGTCGTGGGGCATTTATTGGCGTTGATACGTACAATCGTAATC AGTTAG CS1; TTGAATAAGCTGGTATAAACC CS2; AAACCACCTCCGAATCCCAAGCCACC GCCGCTAACTGATTACGATTGT	CS1; Thiol	-	1.2 (CIP) 1.3 (w) 2.6 (bs) 3.2 (m)	yellow to colorless	12	classic citrate reduction	[83]
OTC	-	CGTACGGAATTCGCTAGCGGGCGGGGGTGCTGGGGGAATGGAGTGCTGCGTGCTGCGGGT CCGAGCTCCACGTG-	-	-	25	red to purple	13	citrate reduction of HAuCl_4_	[84]
OTC	-	CGTACGGAATTCGCTAGCACGTTGACGCTGGTGCCCGGTTGTGGTGCGAGTGTTGTGTGGATCCGAGCTCCACGTG	-	-	01	red to purple	13	citrate reduction of HAuCl_4_	[50]
OTC and KAN	-	OTC apt; CGTACGGAATTCGCTAGCGGGCGGGGGTGTGGGGGAATGGAGTGCTGCGTGCTGCGGGGT CCGAGCTCCACGTG KAN apt; TGG GGG TTG AGG CTA AGC CGA	-	-	1 ag mL^−1^	colorless to blue or yellow	10	citrate reduction of HAuCl_4_	[85]
OTC	-	CGA CGC ACA GTC GCT GGT GCG TAC CTG GTT GCC GTT GTG T	-	-	10 (w) 20 (m)	red to blue	13	Classical citrate reduction	[5]
TET	-	CGTACGGAATTCGCTAGCCCCCCGGCAGGCCACGGCTTGGGTTGGTCCCACTGCGCGTGGATCCGAGCTCCACGTG	-	-	122	purple to red	13	citrate reduction of HAuCl_4_	[86]
TET	-	CGTACGGAATTCGCTAGCCCCCCGGCAGGCCACGGCTTGGGTTGGTCCCACTGCGCGTGGATCCGAGCT CCACGTG	-	-	45.8	red to purple	15	citrate reduction of HAuCl_4_	[86]
TET	-	Apt; CTCTCTCGGTGGTGTCTCTC Signal Transduction Probe; GAGAGAGAGAGAGA	-	-	266 pM	red to blue	15	classical citrate reduction of HAuCl_4_	[87]
TET	-	CGT ACG GAA TTC GCT AGC CCC CCG GCA GGC CAC GGC TTG GGT TGG TCC CAC TGC GCGTGG ATC CGA GCT CCA CGT G	-	-	0.039 µg/mL	red to blue	13	-	[88]
TET	SH–(CH_2_)_6_	CGT ACG GAA TTC GCT AGC CCC CCG GCA GGC CAC GGC TTG GGT TGG TCC CAC TGC GCG TGG ATC CGA GCT CCA CGT G	-	-	0.002 ng/mL,	colorless to blue	18	classical citrate reduction of HAuCl_4_	[89]
SDM	-	SDM apt; GAGGGCAACGAGTGTTTATAGA KAN apt; TGGGGGTTGAGGCTAAGCCGA	-	-	50 ng/m	red to blue	13	Mayer’s method	[90]
SDM	-	GAGGGCAACGAGTGTTTATAGA	-	-	10 ng/mL	red to purplish-blue	13	citrate reduction method	[91]
SDM	-	SDM apt; GGC AAC GAG TGT TTA cDNA; TAA ACA CTC GTT GCC	-	-	3.41 ng mL^−1^ (w) 4.41 ng g^−1^ (f)	red to blue/purple	13	citrate reduction method	[92]

Ampicillin (AMP), Kanamycin (KAN), Tobramycin (TOB), Streptomycin (STR), Neomycin B (NB), Daunomycin (DNR), Chloramphenicol (CAP), Ciprofloxacin (CIP), Oxytetracycline (OTC), Tetracycline (TET), Sulfadimethoxine (SDM), apt = aptamer, cDNA = complementary DNA, FAM = fluorescein amidite, m = milk, dw = distilled water, w = water, f = fish, bs = blood serum, and b = blood.

**Table 3:** Summary of advantages and disadvantages of the reported methods to make a comparison within each antibiotic class and with different classes.

Antibiotic Group/Class	Advantages	Disadvantages
**β-Lactams/(AMP)**	High detection accuracy dual aptasensor with fluorescence and colorimetric approaches proved to be rapid and highly sensitive [59].	-
**Aminoglycosides/** **(KAN), (TOB), (STR), (NB)**	Quick visual readouts (within 3–8 min), high selectivity for quantitative detection of KAN, 15 folds more sensitive and 20 times faster than the conventional aptamer approaches [61]. Rapid, easy, and low-cost detection, visible without any microscope, applications in pharmaceutical preparations and food products [62]. Multiplex detection (3 targets simultaneously). Maintains the same sensitivity as a single-target aptasensor for each individual target by adsorbing more than one class of aptamers onto the surface of AuNPs. Simple design, easy operation, quick response, cost effectiveness and no need for sophisticated instrumentation. Used to screen a variety of samples needed to be screened for multiple antibiotics [63]. Equipment-free, rapid and on-site ultra-sensitive paper chip-based readout using the naked eye. Point-of-care monitoring for food and environmental safety [64]. Multiple incubation and washing steps are avoided. Process completed in 10 min. Lateral flow strip biosensor exhibited high specificity and stability. Detects KAN in various food samples, indicating its great potential in field testing. Qualitative detection by naked eyes or quantitative by a scanning reader [65]. High selectivity and applicability to detect KMC in drinking water and milk samples [67]. Preparation time is short (few minutes). Does not require expensive instruments or a skilled user. Accurate and reliable clinical applications for KAN detection in serum [68]. Quick and cost-effective detection of STR residues in food safety [71]. Point of care testing in food safety [72]. Could be extended to detect biological and environmental samples by replacing corresponding aptamers. Easy to fabricate, favors large-scale production and applications. The accuracy is guaranteed to some extent [73].	Unable to identify individual targets when a sample tests positive [63]. Different food matrices influence the sample composition and sensitivity of detection to a certain extent (e.g., the matrix of milk and milk powder can cause interference in the detection, and the pretreatment can lead to loss of the target to some extent. Thus, the visual detection limits for milk and milk powder samples are higher than that of the standard solution [65]. Preparation process is time consuming and complex [67].
**Anthracyclines/** **(DNR)**	On-site, real-time detection, cost effective and reflection time is only 5 min [76].	-
**Chloramphenicol/** **(CAP)**	Time saving, facile and sensitive than commercial ELISA kits. Can be extended by changing the aptamer sequences to detect ultra-trace level of different antibiotics [78]. More specific, faster and cheaper on site detection [79]. Excellent performance with real samples, sensitivity comparable to electrochemical biosensors. Exhibit high stability and repeatability [80]. Display tolerance to high salt concentrations. Multiplex detection via naked eye, analysis using absorption spectroscopy or smartphone in real samples, cheap, consistent results with desirable recoveries [81]. Portable set-up with strong anti-interference ability and high selectivity [51].	La^3+^ (trigger agent) could limit in-situ applications due to poor biocompatibility and storage instability. Sensitivity and specificity must be further improved [51].
**Fluoroquinolones** **(CIP)**	Flower-shape structure of modified AuNPs warrant that the modified AuNPs do not have any catalytic ability in the absence of target in real samples [83].	-
**Tetracyclines/** **(OTC), (TET)**	In-situ detection via naked eye or UV-vis spectrometer, highly specific and sensitive detection [84]. Reflectance method offers more flexibility in terms of system construction compared with absorbance method. Small amounts of sample and reagents are enough for detection [50]. Rapid, low cost and convenient operation without laborious procedures. It can be extended for the analysis of a wide spectrum of antibiotics by changing the aptamer sequences [85]. Easy-to-build, selective, sensitive and fast detection. Easy realization of material preparation, high selectivity, low detection threshold and high stability of batches [89].	-
**Sulfonamides** **(SDM)**	In-situ detection, simple, fast, and easy to read [90]. Fast, sensitive, cost-effective, reliable and can be extended for other analytes [91].	Only provides a rapid screening of SDM in samples, the results must be rechecked via instrumental methods, such as HPLC-tandem mass spectrometry [92].

## Abbreviations

AuNPs	gold nanoparticles
BSA	bovine serum albumin
BRE	bio-recognition element
cDNA	complementary DNA
CS	complementary strand
dsDNA	double stranded DNA
K_D_	dissociation constant
LOD	limit of detection
SELEX	systematic evolution of ligands by exponential enrichment
SPR	surface plasmon resonance spectroscopy
STE	Signal transduction element
SSB	ssDNA binding protein
ssDNA	single stranded DNA
THMS	triple-helix molecular switch
